# Improving the accuracy of fatty liver index to reflect liver fat content with predictive regression modelling

**DOI:** 10.1371/journal.pone.0273171

**Published:** 2022-09-13

**Authors:** Hykoush A. Asaturyan, Nicolas Basty, Marjola Thanaj, Brandon Whitcher, E. Louise Thomas, Jimmy D. Bell

**Affiliations:** Research Centre for Optimal Health, University of Westminster, London, United Kingdom; University of Campania Luigi Vanvitelli: Universita degli Studi della Campania Luigi Vanvitelli, ITALY

## Abstract

**Background:**

The fatty liver index (FLI) is frequently used as a non-invasive clinical marker for research, prognostic and diagnostic purposes. It is also used to stratify individuals with hepatic steatosis such as non-alcoholic fatty liver disease (NAFLD), and to detect the presence of type 2 diabetes or cardiovascular disease. The FLI is calculated using a combination of anthropometric and blood biochemical variables; however, it reportedly excludes 8.5-16.7% of individuals with NAFLD. Moreover, the FLI cannot quantitatively predict liver fat, which might otherwise render an improved diagnosis and assessment of fatty liver, particularly in longitudinal studies. We propose FLI+ using predictive regression modelling, an improved index reflecting liver fat content that integrates 12 routinely-measured variables, including the original FLI.

**Methods and findings:**

We evaluated FLI+ on a dataset from the UK Biobank containing 28,796 individual estimates of proton density fat fraction derived from magnetic resonance imaging across normal to severe levels and interpolated to align with the original FLI range. The results obtained for FLI+ outperform the original FLI by delivering a lower mean absolute error by approximately 47%, a lower standard deviation by approximately 20%, and an increased adjusted R^2^ statistic by approximately 49%, reflecting a more accurate representation of liver fat content.

**Conclusions:**

Our proposed model predicting FLI+ has the potential to improve diagnosis and provide a more accurate stratification than FLI between absent, mild, moderate and severe levels of hepatic steatosis.

## Introduction

There is a significant rise in the incidence of patients diagnosed with fatty liver, otherwise known as hepatic steatosis, defined as a fat content greater than than 5% of liver weight [1]. Steatosis is characteristic of both alcoholic liver disease (ALD), associated with a high risk of developing alcohol-related hepatitis [2], and non-alcoholic fatty liver disease (NAFLD) [3], associated with the risk of long-term extrahepatic cardiometabolic diseases including heart attack, stroke, type 2 diabetes (T2D) and insulin resistance [4, 5].

The pathophysiological characteristics underlying NAFLD are clinical features of metabolic syndrome and are associated with insulin resistance, which exacerbates NAFLD by increasing hepatic lipogenesis and inhibiting adipose tissue lipolysis [6–8]. NAFLD is prevalent in 25% to 40% of the general population globally [9, 10], rising to a prevalence of 80% to 90% in severely obese populations [11, 12]; as such fatty liver disease is becoming an increasing global public health concern [13, 14]. Multiple studies have demonstrated a strong association between NAFLD and T2D [15, 16], in addition to hyperlipidemia [17], hypertension [18], and an increased risk of developing cardiovascular disease [19, 20] and chronic kidney disease [21, 22].

NAFLD is a progressive condition with its first stage often described as simple steatosis. This leads to inflammation in some individuals, followed by non-alcoholic steatohepatitis (NASH), hepatocyte ballooning, tissue scarring, including fibrosis and cirrhosis, and potentially hepatocellular carcinoma and mortality [5]. The ability to diagnose and characterise hepatic steatosis early helps to stratify patients according to their level of liver fat, assist in decision-making for diagnostic procedures and appropriately target interventions. There have been increased efforts in achieving cost-effective and straightforward diagnostic methods that would be useful for screening, follow-up and evaluating treatment response in clinical practice and research. For example, a FibroScan is a non-invasive device that measures liver “stiffness” using specialised ultrasound technology, which can assess hepatic steatosis and fibrosis [23].

The most commonly applied non-invasive methods used for diagnosing hepatic steatosis include the fatty liver index (FLI) [24], which combines anthropometry with serum a blood test; ultrasound, which is routinely used clinically for grading liver fat accumulation; and more recently, magnetic resonance imaging (MRI) and spectroscopy (MRS). MRI is generally regarded as the gold-standard for the quantitative measurement of liver fat (generally expressed as proton density fat fraction or PDFF). And though MRS is very accurate, it is generally restricted to smaller research studies in specialist centres [25, 26]. While the FLI is best described as a computed score or “predictive probability” for hepatic steatosis, a method such as MRI is required [27–30] to obtain a true quantitative measure of liver fat content and is routinely used in large cohort studies, pharmaceutical and clinical trials [31–33].

For nearly two decades, the clinical and research community have employed the FLI as a surrogate marker for the presence or absence of fatty liver [34–38]. The original FLI algorithm was derived from a study of 216 subjects with NAFLD and 280 controls, with fatty liver diagnosed using abdominal ultrasound. The FLI is calculated using waist circumference, body mass index (BMI), as well as triglycerides and gamma glutamyltransferase (GGT). The FLI has also been used to stratify prediabetes [39], T2D [37], metabolic syndrome [40] and cardiometabolic disease [41, 42].

However, a number of studies have highlighted distinct challenges and drawbacks when employing the FLI to detect the presence of fatty liver in certain subsets of subjects. For example, in a study of 338 volunteers, approximately 8.5% with fatty liver on ultrasound had a normal FLI score, whereas 27.8% of subjects with normal livers, had a FLI suggestive of excess liver fat [43]. Moreover, a recent meta-analysis of 10 different studies [38] comprising of 27,221 subjects showed that the livers of 16.7% of patients were classified as normal despite having confirmed NAFLD, whereas the FLI suggested that 33.3% of patients with normal livers had NAFLD. Furthermore, Cuthbertson *et al.* [44] reported that the FLI could not quantitatively predict liver fat when evaluated against proton MRS (^1^H-MRS) from 336 subjects, 50% of whom had NAFLD.

In recent years, the increasing availability of large MRI datasets from national biobanks has enabled in-depth research towards understanding the impact of diet, lifestyle and genetics on the accumulation and distribution of liver fat [31]. Advancements in artificial intelligence applied to biomedical research have driven the development of regression models that predict body fat using multivariate supervised machine learning methods, such as support vector regression, gradient boosting regression and random forests, all of which are dependent on large datasets (involving more than 100 subjects) [45–50].

In this paper, we propose a regression tool for predicting FLI+, an improved version of the FLI that provides a more accurate reflection of liver fat content. Consequently, FLI+ may be used in clinical practice and research to reduce the number of cases where subjects with fatty liver are assessed as having normal livers and vice-versa, improving the overall stratification between absent, mild, moderate and severe fatty liver.

## Materials and methods

### Data collection of subjects

A dataset involving 28,796 subjects from the UK Biobank imaging cohort was employed (application number 23889). All subjects provided written informed consent, under the UKBB ethical approval from the North West Multi-Centre Research Ethics Committee (MREC). Over 98% of all subjects were of white European ancestry. The age range for inclusion was 44–82 years with varying levels of health status, including subjects who were healthy (≈40%), overweight (≈40%) and obese (≈20%). Additionally, this dataset included 4,913 (≈20%) subjects with at least one feature of metabolic syndrome following the International Diabetes Federation criteria [51]; for example, a waist circumference greater than 94 cm (males) or 80 cm (females), and a combination of T2D and hypertension.

#### Proton density fat fraction

All subjects underwent MRI scanning in a non-fasting state between August 2014 and December 2019 at a UK Biobank imaging centre using a Siemens 1.5T MAGNETOM Aera. A multi-echo spoiled-gradient-echo acquisition was used for the first 10,000 subjects and a single-slice IDEAL sequence was used in the remaining number of subjects [52]. Total acquisition time was less than three minutes with each individual acquisition taking place within a single expiratory breath-hold. No contrast agent was used. The proton density fat fraction (PDFF), expressed as a percentage of liver weight that is fat, was estimated using the PRESCO (Phase Regularized Estimation using Smoothing and Constrained Optimization) algorithm [53] with software available at https://github.com/recoh/pipeline [54].

#### Anthropometric and serum biochemical variables

Anthropometric measurements including weight, height, waist and hip circumferences were taken at the UK Biobank centres. Waist circumference was measured midway between the lower rib margin and the iliac crest, and hip circumference was measured at the greatest protrusion or largest circumference around the buttocks. From the extensive range of blood tests available in the UK Biobank, we selected a range of variables that have shown to be associated with fatty liver, including: gamma glutamyltransferase (GGT), triglycerides, glucose and glycosylated haemoglobin A1c (HbA1c) [55, 56]; white blood cell count [57, 58], uric acid [59, 60], high-density lipoprotein (HDL) [61] and testosterone [62–64]; aspartate aminotransferase (AST), alanine aminotransferase (ALT), and also derived the ratio of AST to ALT [56, 65] and the ratio of AST to platelet count [66, 67]. Diagnoses of liver disease and T2D were obtained from UK Biobank health related outcome data collated from hospital admission records and self-reported sources [68]. The codes are also defined in Text A in S1 File and Table A in S1 File.

### Fatty liver index calculation

The fatty liver index (FLI), as a score between 0 and 100, is defined using BMI (kg/m^2^), serum triglycerides (mg/dL), GGT (U/L) and waist circumference (cm) [24]

$$\begin{array}{c} FLI=\frac{\text{exp}(\lambda )}{1+\text{exp}(\lambda )}\times 100, \end{array}$$ (1)

where

$$\begin{array}{c} \lambda =0.953\times \text{ln}(\text{triglycerides})+0.139\times \text{BMI}+0.718\\ \times \text{ln}(\text{GGT})+0.053\times (\text{waist}\;\text{circumference})-15.745. \end{array}$$ (2)

### Comparing FLI with PDFF

Previous studies have established reference values for both PDFF and FLI that correspond to a normal liver and values relating to elevated or severe liver fat infiltration [27–30, 34–38]. However, a comparison between a quantitative measure such as PDFF and the FLI score, which have entirely different ranges, is not necessarily straightforward. To overcome this challenge and improve the comparison, we performed a linear interpolation for each risk group, mapping the PDFF values for each range to the corresponding FLI range, which is denoted as the Mapped (*M*)-PDFF throughout this paper (Table 1). Additional details are provided in Text B in S1 File.

**Table 1:** MRI derived PDFF risk range mapped to equivalent FLI risk range.

Risk	PDFF	FLI	Mapped PDFF to FLI
Normal	0% ≤ PDFF ≤ 5%	0 ≤ FLI < 30	0 ≤ M-PDFF < 30
Elevated	5% < PDFF ≤ 10%	30 ≤ FLI < 60	30 ≤ M-PDFF < 60
Severe	10% < PDFF ≤ 45%	60 ≤ FLI ≤ 100	60 ≤ M-PDFF ≤ 100

The risk range from PDFF quantifying liver fat compared to the commonly used equivalent risk range for FLI. Mapped (*M*)-PDFF describes the interpolated predictive target range to develop and evaluate FLI+.

### Constructing FLI+ models

We aimed to develop a model for predicting FLI+ in which the ideal target output is *M*-PDFF. To reduce the skewness in the data, it was necessary to apply a log transform to the *M*-PDFF. Consequently, the predicted FLI+ values were exponentially transformed to obtain a score between 0 and 100.

To develop the proposed model, it was essential to select input variables strongly associated with fatty liver. Three different models were developed using “gradient boosting regression” by considering the input variable type and ease of accessibility, with each subsequent model having a reduced number of variables.

#### Gradient boosting regression model

The gradient boosting algorithm [69] is well-known in machine learning, in which the algorithm uses an ensemble of decision trees to minimise the error between a predicted outcome and the expected outcome. A gradient boosting regressor may be used to predict continuous target variables, as in the case of this study.

#### Experimental setup

The proposed method and subsequent analysis was developed using Python 3.7 via Windows 10 running on a GeForce GTX 1060 GPU and i7–8750H CPU at 2.20GHz. The source code is available at https://github.com/pbf-testing/flip. The full dataset was split into training and testing datasets comprising 21,597 (75%) and 7,199 (25%) subjects, respectively. The training and testing datasets share an equal ratio of normal (≈70%), elevated (≈17%) and severe (≈13%) cases of liver fat. The gradient boosting regressor utilised an initial learning rate (0.1) and a loss function of mean squared error (MSE) to minimise the error in every iteration until reaching a pre-specified maximum (100).

### Statistical analysis

All anthropometric characteristics and biochemical variables were initially correlated with PDFF using Pearson’s correlation coefficient. Group comparisons were performed using the Kruskal-Wallis test for categorical variables and a one-way ANOVA for continuous variables. Baseline subject characteristics were reported as median and interquartile range (IQR) for continuous variables, and frequency and percentage for categorical variables. Linear regression with 95% prediction intervals was used to assess the relationship between FLI and PDFF, where the prediction intervals reflect the range of FLI values assigned to a subject for an acquired PDFF.

#### Evaluating FLI+

We aimed to achieve a balance between utilising variables that influence fatty liver while minimising the number of variables that might otherwise have a low impact on accurately predicting FLI+. The percentage of importance in which each of the three gradient boosting regressors selected a variable was analysed. The resultant model predicting FLI+ was evaluated using four-fold cross-validation (CV) to assess performance. That is, the entire dataset was divided into four equal folds, three of which were used for training and the remainder for testing. This process was repeated four different times, after which average measures of error were computed.

To compare FLI+ and the corresponding FLI with the target *M*-PDFF, the mean absolute error (MAE), standard deviation and adjusted coefficient of determination (R^2^) were calculated. To analyse the ability of FLI+ to discriminate between subjects with and without fatty liver at different risk levels, receiver operator characteristic (ROC) curves were constructed with 95% confidence intervals (CIs). The following diagnostic statistics were computed for a range of five-unit intervals: sensitivity (SN), specificity (SP), positive likelihood ratio (LR+) and negative likelihood ratio (LR−).

## Results

### Clinical characteristics of subjects

Subjects were stratified into three groups using liver PDFF cut-off points as previously reported [70]. Participants with PDFF in the normal range were assigned to the normal group, and the remainder were stratified into the elevated or severe groups (see Table 1). The clinical, biochemical variables, anthropometric variables and characteristics of participants are shown in Table 2. Additional information about these variables, and their corresponding relationship with the target PDFF, are provided in Table B in S1 File. The three groups had a comparable median age, but the gender distribution in both the elevated and severe groups was unequal with a higher proportion of male subjects (69.6% and 68.8% respectively). Approximately 29.4% of all 28,796 subjects (*N* = 8, 453) had fatty liver with 5,854 of those being male (69.3%). Moreover, 70.6% (*N* = 20, 343) of all subjects were in the normal group, in which 10,710 were male (52.6%).

**Table 2:** Comparison of anthropometric, biochemical and clinical subject characteristics.

	Normal	Elevated	Severe	***p***-value
*N*	20343	4967	3486	-
Male	10710 (52.6%)	3455 (69.6%)	2399 (68.8%)	< 0.0001
Female	9633 (47.4%)	1512 (30.4%)	1087 (31.2%)	< 0.0001
Age (years)	65 (58, 71)	66 (60, 71)	64 (58, 70)	< 0.0001
Weight (kg)	72.9 (64.1, 82.0)	84.1 (76.0, 93.9)	90.1 (80.6, 100.3)	< 0.0001
BMI (kg/m^2^)	25.1 (23.1, 27.5)	28.4 (26.1, 31.0)	30.4 (27.9, 33.6)	< 0.0001
Waist (cm)	86 (78, 94)	96 (90, 103)	101 (94, 109)	< 0.0001
Hip (cm)	99 (94, 104)	103 (99, 108)	106 (101, 112)	< 0.0001
TG (mg/dL)	23.3 (16.9, 33.0)	32.8 (23.4, 45.4)	36.0 (25.7, 51.5)	< 0.0001
Uric acid (mg/dL)	3.3 (2.8, 3.9)	3.9 (3.3, 4.4)	4.0 (3.4, 4.6)	< 0.0001
Glucose (mg/dL)	87.5 (81.8, 93.5)	89.1 (83.0, 96.2)	90.0 (83.1, 98.5)	< 0.0001
HbA1c (mg/dL)	621.0 (579.6, 662.4)	637.2 (590.4, 682.2)	649.8 (601.2, 707.4)	< 0.0001
HDL (mg/dL)	26.0 (22.0, 31.0)	22.8 (19.5, 27.1)	21.7 (18.6, 25.3)	< 0.0001
TTST (nmol/L)	7.3 (1.0, 12.4)	9.1 (1.5, 12.1)	8.8 (1.6, 11.9)	< 0.0001
GGT (U/L)	23.3 (17.1, 34.6)	32.8 (23.6, 48.0)	37.4 (26.0, 56.8)	< 0.0001
AST (U/L)	23.9 (20.7, 27.9)	25.6 (22.1, 30.4)	27.4 (23.0, 33.6)	< 0.0001
ALT (U/L)	18.9 (14.7, 24.8)	24.3 (19.2, 32.5)	29.4 (21.5, 40.8)	< 0.0001
PLT (10^9^/L)	243.6 (210.6, 280.9)	243.4 (209, 280)	244.5 (211.0, 282.0)	0.5917
WBC (10^9^/L)	6.3 (5.3, 7.4)	6.7 (5.7, 7.7)	6.9 (5.9, 8.1)	< 0.0001
AST:ALT	1.27 (1.04, 1.52)	1.04 (0.86, 1.25)	0.93 (0.77, 1.15)	< 0.0001
AST:PLT	0.10 (0.08, 0.12)	0.11 (0.09, 0.14)	0.11 (0.09, 0.15)	< 0.0001
Waist:Hip	0.87 (0.80, 0.93)	0.93 (0.88, 0.98)	0.95 (0.90, 1.00)	< 0.0001
Type 2 diabetes	889 (4.4%)	584 (11.8%)	699 (20.1%)	< 0.0001
Liver disease	224 (1.1%)	90 (1.8%)	106 (3.0%)	< 0.0001
FLI	8.9 (3.5, 21.5)	32.2 (16.1, 54.5)	49.0 (28.6, 72.6)	< 0.0001
PDFF (%)	2.6 (2.1, 3.4)	6.7 (5.7, 8.0)	14.6 (11.9, 19.2)	< 0.0001

Categorical variables compared using the Kruskal-Wallis test and continuous variables compared using one-way ANOVA. Values are presented as median (interquartile range) for continuous variables and frequency (%) for categorical variables. **Abbreviations**: TG = triglycerides; HDL = high-density lipoprotein cholesterol; HbA1c = glycosylated haemoglobin A1c; TTST = testosterone; GGT = gamma glutamyltransferase; AST = aspartate aminotransferase; ALT = alanine aminotransferase; PLT = platelet count; WBC = white blood cell count; FLI = fatty liver index; PDFF = proton density fat fraction.

Subjects with fatty liver were primarily overweight or obese, with significantly higher BMI and waist-to-hip ratio compared to subjects with normal levels of PDFF. They exhibited statistically higher (*p* < 0.0001) serum uric acid with a median of 3.9 mg/dL (IQR 3.3–4.5 mg/dL) versus 3.3 mg/dL (IQR 2.8–3.9 mg/dL), glucose with a median of 89.4 mg/dL (IQR 83.0–97.1 mg/dL) versus 87.5 mg/dL (IQR 81.8–93.5 mg/dL), GGT with a median of 34.6 U/L (IQR 24.4–51.6 U/L) versus 23.3 U/L (IQR 17.1–34.6 U/L) and triglycerides with a median of 34.0 mg/dL (IQR 24.4–48.4 mg/dL) versus 23.3 mg/dL (IQR 16.9–33.0 mg/dL). All three groups had a comparable rate of T2D, but the liver disease diagnosis was approximately two and five times higher in the elevated and severe group compared to the normal group, respectively.

**Fig 1:**
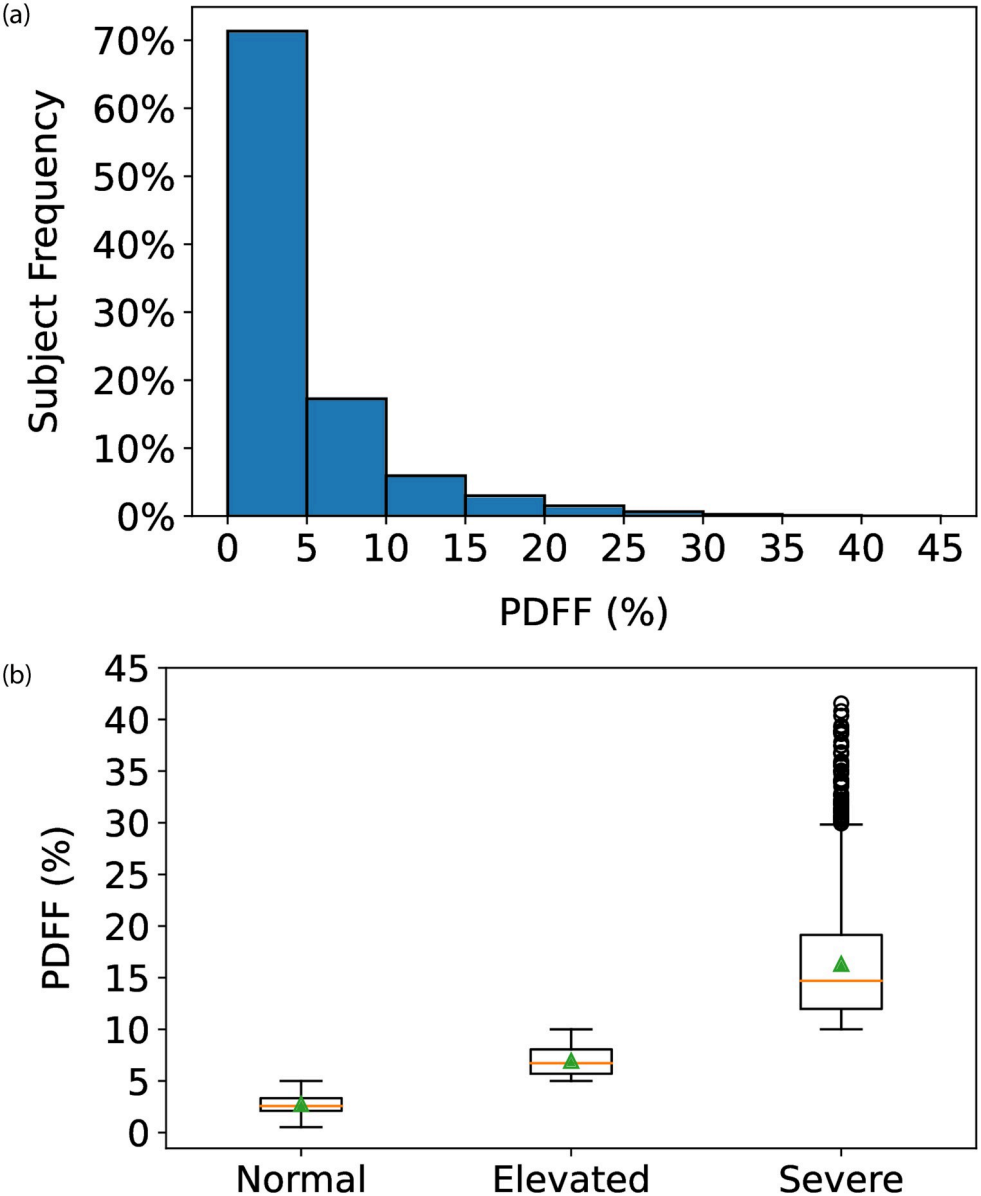
Proton density fat fraction (PDFF) in (a) all subjects and (b) three groups.

Given how the groups were constructed, the PDFF (%) was significantly lower (*p* < 0.0001) in the normal group (median 2.6%, IQR 2.1–3.4%) compared to the elevated (median 6.7%, IQR 5.7–8.0%) and severe (median 14.6%, IQR 11.9–19.2%) groups. Fig 1(a) displays a distribution of PDFF across all subjects and Fig 1(b) indicates the amount of variation in PDFF for each of the three groups provided in Table 2. Differences in PDFF were reflected in the serum biochemical results with significantly higher AST, ALT, GGT and triglycerides, and lower HDL cholesterol in the elevated and severe groups. Subjects within a normal PDFF range had significantly lower FLI with a median of 8.9 (IQR 3.5–21.5) compared to 32.2 (IQR 16.1–54.5) and 49.0 (IQR 28.6–72.6) in the elevated and severe groups, respectively.

### Correlating FLI with PDFF

**Fig 2:**
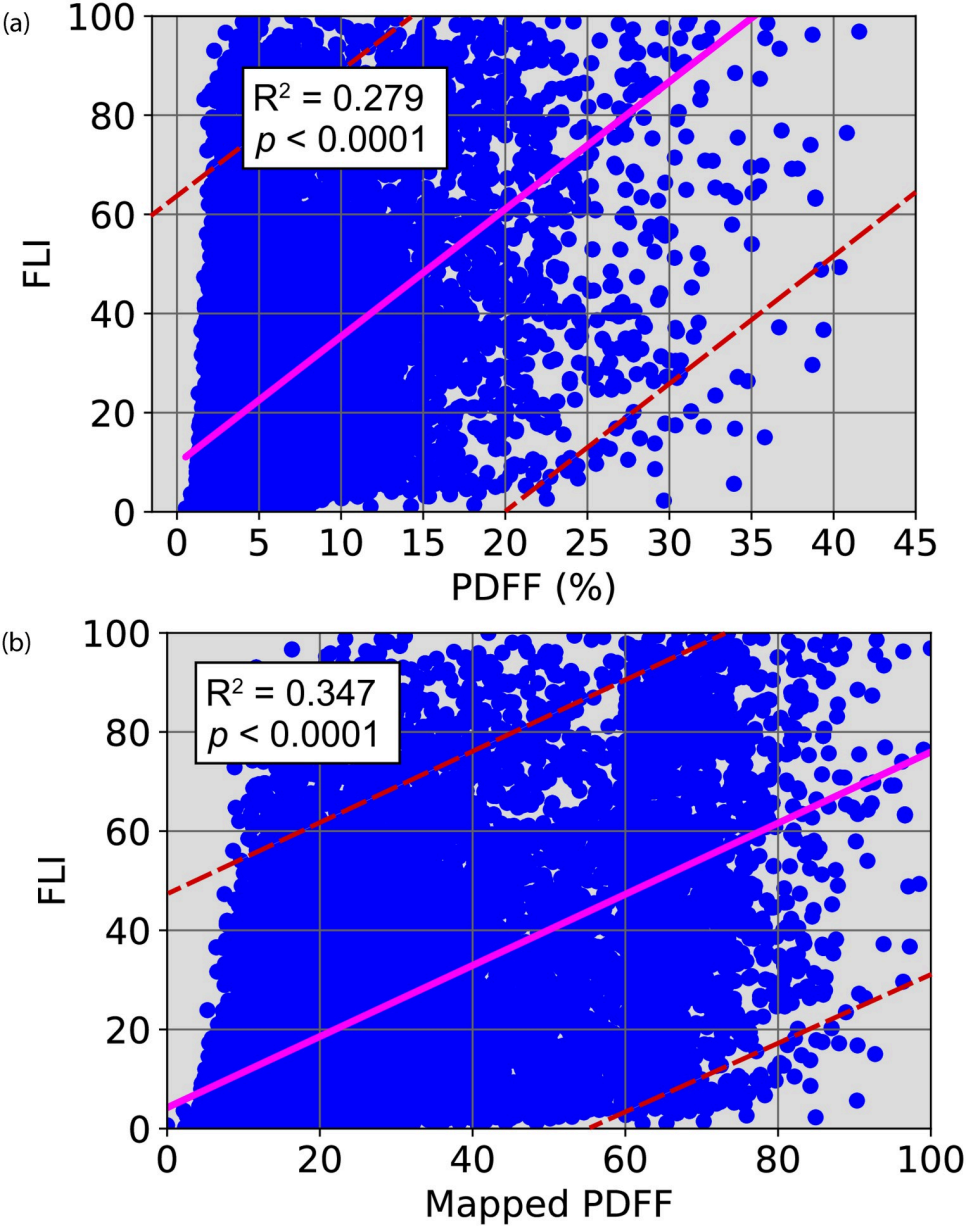
(a) Correlation of fatty liver index (FLI) against respective proton density fat fraction (PDFF); (b) Relationship between FLI and the mapped PDFF. (a) FLI versus PDFF. (b) FLI versus mapped PDFF.

The relationship between the FLI and corresponding PDFF for all subjects is shown in Fig 2(a) (R^2^ = 0.279). Taking into account the interpolated PDFF values for each range to the corresponding FLI range for a particular risk group, the variation in FLI against the *M*-PDFF (R^2^ = 0.347) is shown in Fig 2(b).

### Feature importance

**Fig 3:**
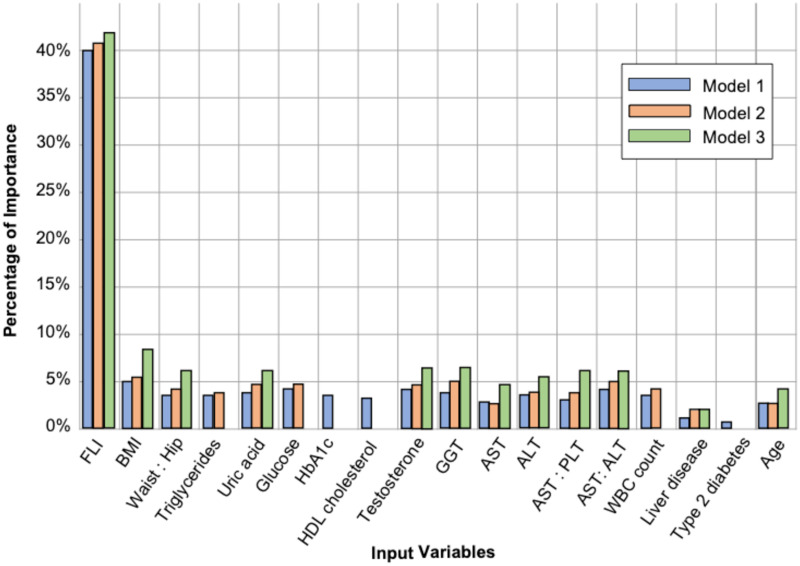
Input variable importance in the three models.

Input variable importances for the models are shown in Fig 3. The FLI had a significant impact on each model’s output. Model 1 integrated all 18 variables while Model 2 excluded T2D status, HbA1c and HDL cholesterol to mitigate opposite serum levels in subjects with NAFLD and ALD. Model 3 further excluded serum biochemistry variables that did not significantly impact the resultant R^2^ statistic, MAE or SD, including white blood cell (WBC) count, glucose and triglycerides as an independent variable albeit integrated into the computation of FLI. Thus, Model 3 was selected as the resultant regression model to predict FLI+ by integrating 12 input variables including FLI (41%), BMI (7.5%), the waist-to-hip ratio (5.9%), uric acid (6.0%), testosterone (6.4%), GGT (6.3%), AST (4.5%), ALT (5.6%), AST to ALT ratio (5.7%), AST to platelet count ratio (5.7%), liver disease diagnosis (1.0%) and age (4.4%).

### Performance of FLI+

The MAE, SD and adjusted R^2^ statistic for FLI+ and corresponding FLI were evaluated against the target *M*-PDFF across a four-fold CV to assess the robustness in the proposed gradient boosting model’s performance (Table 3). There was a slight variation for each fold (SD 0.01 to 0.16) for FLI+, as shown in the second to the fourth columns. The FLI+ outperformed the original FLI by delivering a lower MAE by approximately 47%, a lower SD by approximately 20% and an increased adjusted R^2^ statistic by approximately 49%, reflecting a more accurate representation of liver fat content.

**Table 3:** Performance of FLI+ versus FLI.

CV-Fold	FLI+			FLI		
	MAE	SD	R^2^	MAE	SD	R^2^
1	7.67	10.5	0.557	14.3	13.2	0.372
2	7.87	10.7	0.535	14.8	12.9	0.369
3	7.61	10.4	0.552	14.4	13.2	0.371
4	7.83	10.8	0.520	14.7	13.8	0.334
**Mean**	7.75	10.6	0.541	14.6	13.3	0.362

Four-fold cross-validation (CV) results of mean absolute error (MAE), standard deviation (SD) and coefficient of determination (R^2^) of fatty liver index (FLI)+ and FLI when compared to mapped proton density fat fraction (PDFF) interpolated to FLI range and threshold.

**Fig 4:**
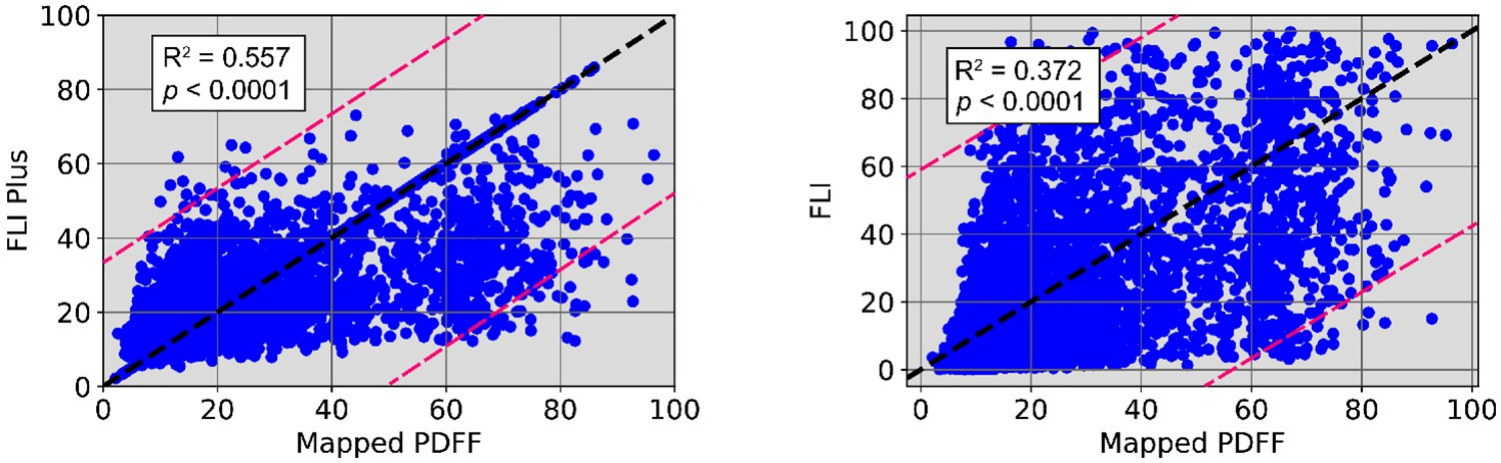
Correlation of fatty liver index (FLI) Plus (a) FLI (b) with proton density fat fraction (PDFF) mapped to the FLI risk range and threshold. (a) FLI+ versus mapped PDFF. (b) FLI versus mapped PDFF.

The 95% prediction intervals between FLI+ and *M*-PDFF for all test subjects for one CV fold (R^2^ = 0.557) is shown in Fig 4(a), while Fig 4(b) provides insight between the corresponding FLI and *M*-PDFF in the same CV fold (R^2^ = 0.372). Notice that for a *M*-PDFF of 40, the resultant FLI values overflowed the entire 0–100 range, whereas FLI+ resulted in a tighter predicted range of 10–70. Moreover, FLI+ outperformed FLI between a *M*-PDFF of 40–100 in the upper prediction interval region. Moreover, FLI+ achieved an improved score in both the upper and lower prediction intervals for a *M*-PDFF between 0–40 compared to FLI. For example, for a *M*-PDFF of 20, FLI spanned 0–98, whereas FLI+ delivered a more accurate and conservative range of 10–65. Another method of comparison using Bland-Altman plots is provided in Fig A in S1 File.

To assess the performance of FLI+ in every risk group, we compared the average MAE and SD (Table 4) and found a higher MAE in the elevated and severe groups by approximately two to five times compared to the normal group, with comparable SD across all three groups. There was a higher degree of accuracy in the upper interval for the severe group between approximately 40 and 80 *M*-PDFF as shown in Fig 4(a).

**Table 4:** Mean performance of FLI+ and FLI stratified by risk groups.

Risk	MAE		SD	
	FLI+	FLI	FLI+	FLI
Normal	4.80	11.0	5.92	10.6
Elevated	10.6	20.9	10.5	13.4
Severe	21.5	25.8	17.9	16.8

FLI+ achieved an improvement in MAE that was approximately two times lower compared to FLI in the elevated and normal groups with 10.6 versus 20.9 and 4.80 versus 11.0, respectively. FLI+ also achieved an improved MAE in the severe group that is lower by approximately 0.8 compared to FLI, suggesting the relatively higher variation in PDFF shown in Fig 1(b) might have impacted the robustness of the prediction model. Table 5 shows for each risk group the average percentage of FLI+ having a score with a lower absolute error than its corresponding FLI. The severe group achieved a more accurate prediction in 58.1% of subjects, whereas the normal and elevated groups achieved a more accurate prediction in more than 77.0% of subjects.

**Table 5:** Percentage of predicted FLI+ values with lower absolute error compared to original computed FLI.

Risk	Mapped PDFF to FLI	FLI+
Normal	0 ≤ M-PDFF < 30	77.7%
Elevated	30 ≤ M-PDFF < 60	78.0%
Severe	60 ≤ M-PDFF ≤ 100	58.1%

**Fig 5:**
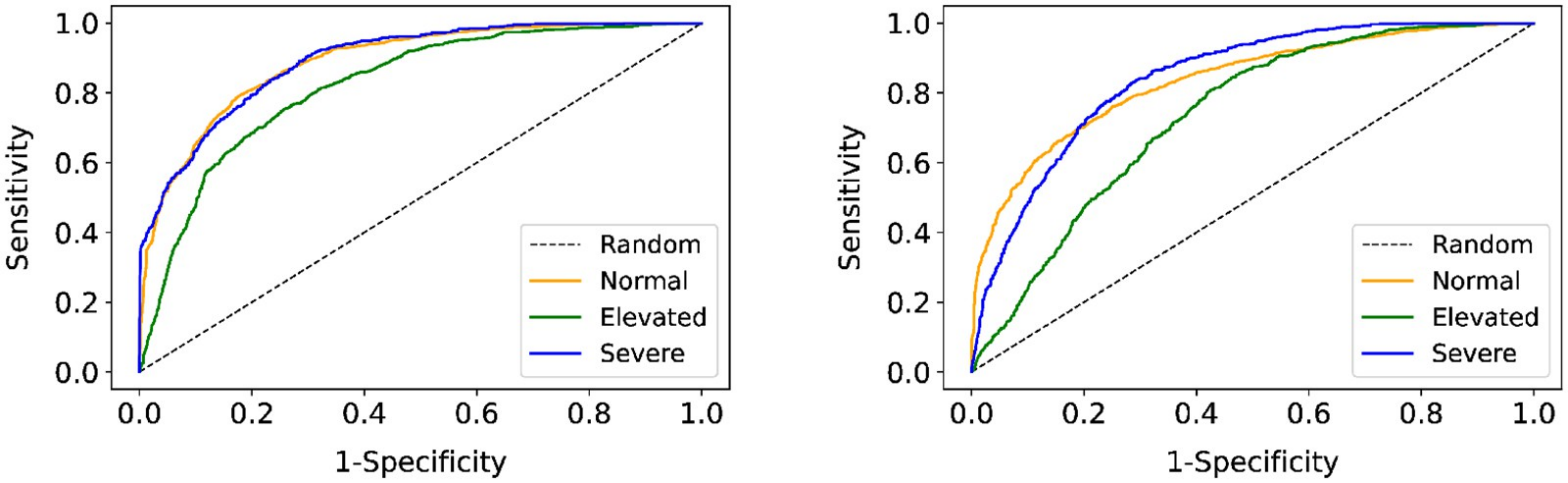
Receiver operating characteristic (ROC) curves for (a) FLI+ and (b) FLI to distinguish the presence or absence of fatty liver. Normal group represents non-fatty liver whereas elevated and severe represent fatty liver. (a) FLI+. (b) FLI.

Receiver operator characteristic (ROC) curves were constructed for each of the three groups (normal, elevated and severe) as shown in Fig 5(a). The average area under the ROC curve (AUROC) resulted in 0.86 (95%CI 0.79,0.91) and highlights the ability of FLI+ to discriminate between subjects with and without fatty liver. By comparison, Fig 5(b) illustrates that FLI resulted in an AUROC of 0.79 (95%CI 0.72,0.84).

The diagnostic accuracy of FLI+ to predict fatty liver by five-unit intervals, as per the original study [24], is provided in Table 6(a) and reports the percentage of subjects (%), SN, SP, LR+ and LR−. For example, a cut-off point of FLI+ ≥ 10 resulted in an LR− of 0.25, indicating a four-fold decrease in the odds of having fatty liver given a negative test result. Moreover, the same cut-off point was moderate at ruling in fatty liver given a positive test result with an LR+ of 6.94.

**Table 6:** FLI+ and FLI cut-point table.

**CP**	**%**	**SN**	**SP**	**LR+**	**LR−**
≥10	91.2	0.78	0.89	6.94	0.25
≥20	45.9	0.65	0.82	3.68	0.43
≥30	24.4	0.55	0.77	2.41	0.59
≥40	13.0	0.64	0.82	3.62	0.43
≥50	7.19	0.79	0.89	7.33	0.24
≥60	4.22	0.93	0.97	27.6	0.07
(a) FLI+					
**CP**	**%**	**SN**	**SP**	**LR+**	**LR−**
≥10	57.2	0.58	0.79	2.79	0.53
≥20	39.3	0.50	0.75	2.00	0.67
≥30	28.7	0.44	0.72	1.60	0.77
≥40	20.8	0.42	0.71	1.44	0.82
≥50	15.0	0.40	0.70	1.34	0.86
≥60	9.97	0.43	0.72	1.53	0.79
(b) FLI					

Fatty liver index (FLI)+ (a) and FLI (b) cut-point table by five-unit intervals. Abbreviations: CP = cut-point; % = proportion of patients with an FLI+ and FLI cut-point; SN = sensitivity; SP = specificity; LR+ = positive likelihood ratio; LR− = negative likelihood ratio.

The corresponding predictive scores of FLI scores are shown in Table 6(b), where an FLI+ and FLI cut-point ≥10 had a sensitivity of 0.78 versus 0.58, respectively. An FLI+ cut-point ≥ 60 resulted in a specificity of 0.97 versus 0.72 in FLI and a significantly different LR+ of 27.6 versus 1.53, respectively. In other words, given a cut-off point of FLI+ ≥ 60, an LR+ of 27.6 indicates a 28-fold increase in the odds of ruling in fatty liver given a positive test result. An LR− of 0.07 delivered a 14-fold decrease in the odds of having excess liver fat given a negative test result.

### Application of FLI+ to an additional UK Biobank dataset

To further compare the outcome between FLI+ and FLI, we applied our methods to the wider UK Biobank cohort who were not part of the imaging sub-study (*N* = 373, 255). Since these participants had not undergone imaging, no liver PDFF measurements were available. This cohort was aged 37–73 years, with an approximately equal proportion of male and female subjects, of whom 32% were in the healthy BMI range, 43% were overweight and 25% were obese. The FLI algorithm resulted in a median score of 16.7 (IQR 5.5–41.5), whereas the model predicting FLI+ delivered a median of 21.4 (IQR 14.3–32.5). A summary of the anthropometric, biochemical and clinical subject characteristics are provided in Table C in S1 File and additional supporting information is provided in Text C in S1 File, Tables D–F in S1 File.

## Discussion

This paper presents a predictive regression model using a gradient boosting algorithm to calculate FLI+ as a score between 0 and 100, reflecting a subject’s liver fat level. The proposed model was rigorously evaluated using four-fold cross validation, in which the predicted FLI+ outperformed the original FLI with an increased R^2^ statistic and significantly lower mean absolute error and standard deviation, demonstrating consistency in performance with potential for clinical translation.

The proposed predictive model utilised a UK Biobank dataset containing 28,796 estimates of MRI-derived PDFF across normal to severe levels, and integrated the following input variables: age, the waist-to-hip ratio, BMI, original computed FLI, uric acid, testosterone, GGT, AST, ALT, the ratio of AST to ALT, the ratio of AST to PLT and liver disease diagnosis.

We have shown that approximately 4% of all subjects evaluated in the context of this study had an FLI+ ≥ 60, whereas approximately twice as many had the same FLI cut-point. Similarly, approximately 10% of subjects had an FLI+ < 10, whereas approximately 40% had the same FLI cut-point. In this study, a subject without fatty liver was approximately four times more likely to have an FLI+ < 10, and a subject with fatty liver was approximately 28 times more likely to have an FLI+ ≥ 60. In contrast, a subject without fatty liver was approximately two times more likely to have an FLI < 10 and a subject with fatty liver was approximately 1.5 times more likely to have an FLI ≥ 60. Considering the original study that derived the FLI algorithm using abdominal ultrasound [24], the ground-truth used in the development of a regression model predicting FLI+ was MRI-derived PDFF estimates of liver fat content. We note that the original study had a sensitivity of 0.55 for predicting in favour of fatty liver at a FLI ≥ 30, while FLI+ had a lower sensitivity by approximately 1.5 times. However, if the cut-off point of the FLI was ≥60 to rule in favour of fatty liver at a sensitivity of 0.61, as in the original study, the sensitivity for FLI+ increased to 0.93.

By evaluating our predictive model, the FLI+ score discriminates between subjects with normal and elevated or severe fatty liver (≥5%) and achieves an AUROC of 0.86. We have also shown that the FLI+ score compared to FLI is 77.7% more accurate in predicting normal liver and 78.0% and 58.1% more accurate in predicting elevated and severe fatty liver, respectively.

The FLI has been previously examined against gold-standard measurements of quantitative liver fat derived from MRI in the last decade. However, these studies involved relatively small cohorts, often characterised by a specific gender or health status. For example, Bozkurt et al. [71] reported a study involving 26 female subjects, 17 diagnosed with previous gestational diabetes (pGDM) and eight with normal glucose tolerance during pregnancy, all of whom underwent ^1^H-MRS and demonstrated a strong nonlinear relationship between FLI and liver fat content with limited predictive ability. Another study involving ^1^H-MRS and 92 non-diabetic, predominantly non-obese subjects achieved AUROC = 0.72 for the FLI and related positively to fatty liver [72]. Using ^1^H-MRS to measure liver fat as the gold-standard, the discriminative ability of FLI was demonstrated in Cuthbertson et al. [44] involving 168 subjects with NAFLD and 168 healthy controls with AUROC = 0.79; however, the FLI could not quantitatively predict liver fat. Interestingly, the inclusion of a dual-echo chemical shift imaging MRI technique in a more extensive study involving 392 and 909 subjects with and without NAFLD subjects, respectively, reported a lower diagnostic performance of AUROC = 0.68 for FLI [73].

As a non-invasive method, predicting FLI+ can improve stratification between absent, mild, moderate and severe fatty liver in a large cohort of subjects without the expensive costs of frequent MR imaging. Thus, researchers that aim to investigate changes in accumulated liver fat due to interventions in diet or exercise can integrate the FLI+ score as a valuable marker to screen for the presence of or severity of fatty liver throughout a clinical study.

It is worth pointing out that the blood chemistry tests in the UK Biobank were performed at the initial assessment visit, and the MRI was performed during subsequent visits. Furthermore, whereas the original study introducing the FLI algorithm integrated blood markers measured after eight hours of fasting, a limitation of this study is that only non-fasted blood samples were available in the UK Biobank. Thus, although we analysed glucose levels in the context of this study, we also analysed the HbA1c marker, which does not require fasting. Previous research has shown that results obtained from non-fasting versus fasting glucose in large-scale studies are not statistically significant. Moreover, a recently published study involving 8,270 subjects showed excellent 94.8% agreement between fasting and non-fasting cholesterol levels [74]. We recognise that non-fasting triglycerides, GGT and testosterone measures [75, 76] could impact the predictive modelling outcome depending on the period lapsed between a participant’s blood test and their last intake of high carbohydrates.

Indeed, we also acknowledge that the FLI+ indicates the level of accumulated fat in the liver without discriminating between ALD, NAFLD or metabolic dysfunction in NAFLD. Moreover, we recognise that the level of liver fat does not always correspond to disease severity. For example, individuals with NASH-related cirrhosis have a reduced quantitative liver fat content than those with early-stage NAFLD. Consequently, the FLI+ score can integrate into a medical study or assessment alongside the individual’s dietary factors and possible external symptoms that might support characterising their condition.

An additional limitation of this study is the large imbalance of genetic ancestry groups in which over 98% of subjects were white European. Future work might seek to obtain and integrate data from a variety of subject demographics, including African, Central and South Asian and East Asian ancestry. We also acknowledge that this study has an imbalance in the proportion of subjects having fatty liver compared to non-fatty. Future work could explore techniques to counter the data imbalance when developing a model to predict a more accurate FLI reflective of a subject’s level of liver fat. However, this study had the advantage of utilising an MRI-PDFF dataset of 28,796 subjects with normal liver fat and varying levels of fatty liver that reflects the general population. While this study using FLI achieved AUROC comparable to previous works, FLI+ consistently improved the discriminative ability across all risk levels.

Future research will compare the FLI+ with available indices that have been previously derived to identify subjects with a form of fatty liver as inferred using ^1^H-MRS or ultrasound, including the NAFLD liver fat score [77], Hepatic steatosis index (HSI) [78], ZJU index [79], Framingham steatosis index (FSI) [80] and the Dallas steatosis index (DSI) [81]. Although these indices were derived initially using fasting blood markers, a recent study performed an external validation on the DSI in the UK Biobank cohort [82] and reported that while the DSI does not discriminate between subjects with NAFLD and those with NASH, it is a valid algorithm to predict NAFLD. The FSI and DSI also integrate information about a subject’s diagnosis of hypertension and diabetes, and the NAFLD liver fat score requires diagnosis relating to metabolic syndrome, unlike the FLI+ score that is primarily based on anthropometry, blood markers and liver disease diagnosis.

Subsequent work will also investigate deep learning regression techniques to increase the prediction accuracy closer to the quantitative level of liver fat. Depending on the nature of a clinician’s workflow, it may be preferable to utilise a model that predicts a subject’s category of liver fat. Thus, future work may build upon the regression model to introduce a classification model that predicts a single category of normal, elevated or severe fatty liver.

## Conclusions

By integrating the most extensive MRI dataset from the UK Biobank, we developed a regression model that predicts FLI+ as an improvement to FLI in a large cohort reflective of the general adult population. Obtaining an FLI+ score requires four basic anthropometric measurements and a standard set of seven biochemical blood variables readily obtainable in clinical and research settings.

The FLI+ has the potential to improve diagnosis and provide a more accurate stratification than FLI between absent, mild, moderate and severe levels of hepatic steatosis. In addition, the model predicting FLI+ can be easily used as a standalone tool or integrated into a more extensive computational system for research or clinical purposes.

## Supporting information

S1 File This file contains the supplementary information: Texts A–C, Tables A–F, and Figs A–C. (PDF)
