# Supplementary material for: Improving the accuracy of fatty liver index to reflect liver fat content with predictive regression modelling
Source: PLoS One. 2022 Sep 13;17(9):e0273171. doi: 10.1371/journal.pone.0273171 (PMC9469950; doi:10.1371/journal.pone.0273171)
Supplement: S1 File — (PDF) [file pone.0273171.s001.pdf]

Improving the accuracy of fatty liver index to reflect liver fat content with predictive regression modelling

Hykoush A.Asaturyan[1], Nicolas Basty[1], Marjola Thanaj[1], Brandon Whitcher[1], E. Louise Thomas[1], Jimmy D. Bell[1*]

1 Research Centre for Optimal Health, University of Westminster, London, UK

* J.Bell@westminster.ac.uk

Supporting information

Text A

The variables describing a medical condition, including liver disease and T2D, were established by self-reported information via UK Biobank codes or recorded using hospital episode statistics (HES). A subject was classified as a "case" if they were registered in HES data using the International Classification of Diseases (ICD) 9th and 10th edition or self-reported codes at least once before their first imaging visit.

Table A

Table S1 lists the corresponding codes for the categories of medical conditions. An empty cell indicates there is no code for a particular category. The International Classification of Diseases (ICD) codes for type 2 diabetes were based on the ICD codes for "non-insulin-dependent diabetes mellitus" and the self-reported code for "diabetes" and "type 2 diabetes". The ICD codes for liver disease were taken from Schneider *et al.* [1] and the self-reported codes used were "liver failure/cirrhosis", "infective/viral hepatitis", "alcoholic liver disease/alcoholic cirrhosis", and "liver/biliary/pancreas problem".

Table S1. Code definitions for medical conditions

Trait	SNOMED	ICD9	ICD10	Field 20002	Field 20001	Other Fields
Liver disease (defined by chronic viral hepatitis, malignant liver disease, NAFLD and hepatic cirrhosis)	235856003 3738000 197315008 19943007	0700|0702|0703|0704|0709|155|1551|5715|57151|5716|5718|573|5731|5734	B18|C22|K70-K77	1136|1156|1158|1604	cell6 row 2	cell7 row 2
Type 2 diabetes mellitus	44054006	25000	E11|E110-E119	1220|1223		

Assigned definitions for medical conditions of liver disease and type 2 diabetes mellitus. Field 20001: self-reported cancer illness; Field 20002: self-reported non-cancer illness; ICD9: International Classification of Diseases 9th edition; ICD10 International Classification of Diseases 10th edition.

Text B

A dataset containing N proton density fat fraction values (PDFF) within a category risk range $[\,a,\, b\,]$ can be interpolated using linear polynomials to construct a set of N mapped or M-PDFF values within the corresponding fatty liver index (FLI) range or interval $[\,c,\, d\,]$.

Supposing that dataset $\mathbf{PDFF} = \{\mathrm{PDFF}_i, ..., \mathrm{PDFF}_N\}$ where $i \in \mathbb{Z}^+ : 1 \leqslant i \leqslant N$, we can construct the dataset $\mathbf{M\text{-}PDFF} = \{M\text{-}\mathrm{PDFF}_i, ...M\text{-}\mathrm{PDFF}_N\}$ using Equation S2.

$$\mathbf{M\text{-}PDFF} = c + \left(\frac{d-c}{b-a}\right)(\mathbf{PDFF} - a) \tag{S2}$$

Table B

Table S2 displays the clinical, biochemical and anthropometric variables of interest included in the development and evaluation of the model predicting fatty liver index plus (FLI+), and the corresponding relationship with the target liver proton density fat fraction (PDFF).

Table S2. Subject variables of interest.

	r	***p*-value**
Age (years)	-0.0114	< 0.0001
Weight (kg)	0.4395	< 0.0001
BMI (kg/m^2)	0.4766	< 0.0001
Waist (cm)	0.4638	< 0.0001
Hip (cm)	0.3434	< 0.0001
TG (mg/dL)	0.2877	< 0.0001
Uric acid (mg/dL)	0.2819	< 0.0001
Glucose (mg/dL)	0.1042	< 0.0001
HbA1c (mg/dL)	0.1514	< 0.0001
HDL (mg/dL)	-0.2611	< 0.0001
TTST (nmol/L)	0.0758	< 0.0001
GGT (U/L)	0.1953	< 0.0001
AST (U/L)	0.1777	< 0.0001
ALT (U/L)	0.3391	< 0.0001
PLT (10^9/L)	-0.0132	< 0.0001
WBC (10^9/L)	0.1171	< 0.0001
AST:ALT	-0.2972	< 0.0001
AST:PLT	0.1350	< 0.0001
Waist:Hip	0.3613	< 0.0001
Liver disease	0.1442	< 0.0001
Type 2 diabetes	0.2088	< 0.0001
FLI	0.5283	< 0.0001
PDFF (%)	-	-

Pearson's correlation coefficient (r) and corresponding p-value is shown with respect to the liver proton density fat fraction (PDFF). **Abbreviations:** TG = triglycerides; HDL = high-density lipoprotein cholesterol; HbA1c = glycosylated haemoglobin A1c; TTST = testosterone; GGT = gamma glutamyl transpeptidase; AST = aspartate transaminase; ALT = alanine transferase; PLT = platelet count; WBC = white blood cell count; FLI = fatty liver index.

Figure A

Fig S3 (a) displays the Bland-Altman plot of computed fatty liver index (FLI)+ values using a test dataset (7,199 subjects) and corresponding mapped proton density fat fraction values or M-PDFF. Fig S3 (b) displays the plot of corresponding predicted FLI values and M-PDFF. In each plot, the thick horizontal line represents the mean difference and the red dotted horizontal lines represent the limits of agreement.

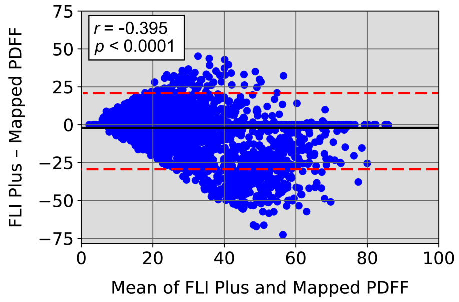

(a) FLI+

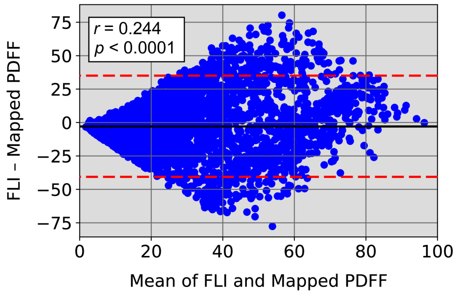

(b) FLI

Fig S3. **Bland-Altman plots of (a) fatty liver index (FLI)+ and Mapped proton density fat fraction (PDFF) and (b) corresponding FLI and Mapped PDFF. Thick horizontal line represents mean difference and hotted horizontal lines represent limits of agreement defined as the mean difference $\pm 1.96 \times$ standard deviation.**

The plot for FLI+ in Fig S3 (a) displays a statistically significant correlation between the mean FLI+ and M-PDFF values and the difference between FLI+ and M-PDFF ($r = -0.395$). The plot for the corresponding FLI in Fig S3 (b) displays a statistically significant positive correlation ($r = 0.244$).

Text C

We utilised a non-imaging cohort in the UK Biobank consisting of 373,255 subjects with no available liver proton density fat fraction (PDFF) values to further compare the outcome between fatty liver index (FLI) and FLI+. A summary of the subject characteristics are presented in Table S4.

The computed indices can be stratified into three risk groups: normal (< 30); elevated (≥ 30 and < 60); and severe (≥ 60). A summary of the results are presented in Tables S5, S6 and S7.

Table C

Table S4. Subject characteristics of non-imaging UK Biobank cohort.

Variable	Non-imaging cohort
N	373,255
Male	187,822 (50.3%)
Female	185,433 (49.7%)
Age (years)	58 (50, 63)
Weight (kg)	77.4 (67.5, 88.4)
BMI (kg/m^2)	26.8 (24.3, 29.9)
Waist (cm)	91 (81, 99)
Hip (cm)	102 (98, 108)
TG (mg/dL)	26.9 (18.9, 38.9)
Uric acid (mg/dL)	3.5 (2.9, 4.1)
TTST (nmol/L)	4.6 (1.0, 11.6)
GGT (U/L)	26.8 (18.8, 41.7)
AST (U/L)	24.5 (21.1, 29.0)
ALT (U/L)	20.4 (15.6, 27.8)
PLT (10^9/L)	247.0 (212.5, 286.0)
AST:ALT	1.2 (0.96, 1.45)
AST:PLT	0.1 (0.07, 0.13)
Waist:Hip	0.88 (0.81, 0.94)
Liver disease	756 (0.20%)

Values are presented as median (interquartile range) or frequency (%). **Abbreviations:** N = number of subjects; TG = triglycerides; TTST = testosterone; GGT = gamma glutamyltransferase; AST = aspartate aminotransferase; ALT = alanine aminotransferase; PLT = platelet count;

Table D

Table S5. General measures of FLI+ and FLI using non-imaging UK Biobank cohort.

	N	FLI+	FLI
All subjects	373,255	21.4 (14.3, 32.5)	16.7 (5.5, 41.5)
Male	187,822	25.3 (17.7, 36.0)	27.1 (11.9, 52.4)
Female	185,433	17.3 (12.2, 27.7)	8.4 (3.0, 25.7)

FLI+ and FLI values are presented as median (interquartile range) using data from a non-imaging UK Biobank cohort. **Abbreviations:** N = number of subjects; FLI = fatty liver index.

Table E

Table S6. Stratified measures of FLI+ and FLI using non-imaging UK Biobank cohort.

Subjects	Risk	FLI+	FLI
All	Normal	16.9 (12.7, 22.5)	8.1 (3.5, 16.3)
	Elevated	38.8 (33.9, 45.4)	42.6 (35.9, 50.5)
	Severe	64.6 (62.0, 68.3)	76.7 (67.7, 86.9)
Male	Normal	19.5 (15.2, 24.3)	12.8 (6.8, 20.3)
	Elevated	38.9 (34.1, 45.8)	42.9 (36.1, 50.7)
	Severe	64.7 (61.9, 68.3)	76.4 (67.6, 86.5)
Female	Normal	14.7 (11.4, 20.2)	5.4 (2.4, 12.1)
	Elevated	38.5 (33.8, 44.7)	42.1 (35.5, 50.1)
	Severe	64.6 (62.1, 68.3)	77.3 (67.9, 87.7)

Values are presented as median (interquartile range) according to subjects stratified into risk groups based on FLI+ and FLI values in a non-imaging UK Biobank cohort. **Abbreviations:** N = number of subjects; FLI = fatty liver index.

Table F

Table S7. Percentage of non-imaging UK Biobank cohort stratified into risk groups.

Subjects	Risk	FLI+	FLI
All	Normal	70.4%	65.7%
	Elevated	26.4%	19.9%
	Severe	3.20%	14.4%
Male	Normal	62.4%	53.5%
	Elevated	33.3%	26.8%
	Severe	4.30%	19.7%
Female	Normal	78.5%	77.9%
	Elevated	19.5%	13.1%
	Severe	2.00%	9.00%

Percentage (%) of subjects stratified into risk groups according to FLI+ and FLI using a dataset from a non-imaging UK Biobank cohort. **Abbreviations:** FLI = fatty liver index.

Figure B

We further analysed additional data from a non-imaging cohort in the UK Biobank introduced in Section Text C, such that Figs S8(a), (b) and (c) illustrates the distribution of fatty liver index (FLI) and FLI+ across all subjects, male subjects and female subjects, respectively.

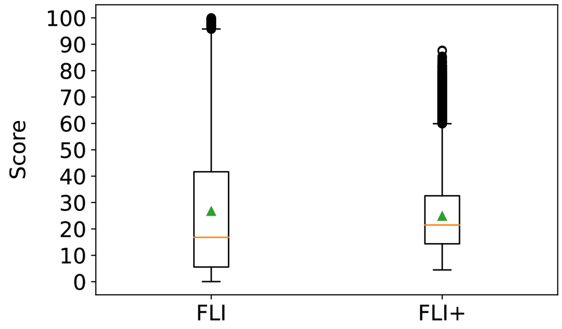

(a) All subjects

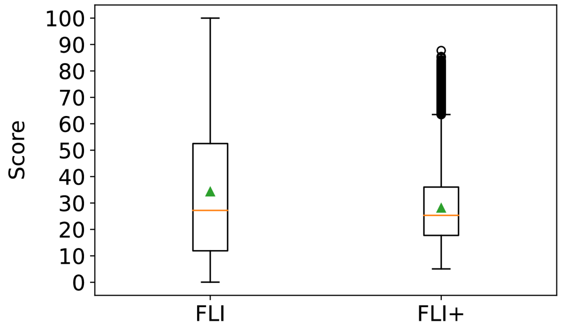

(b) Male subjects

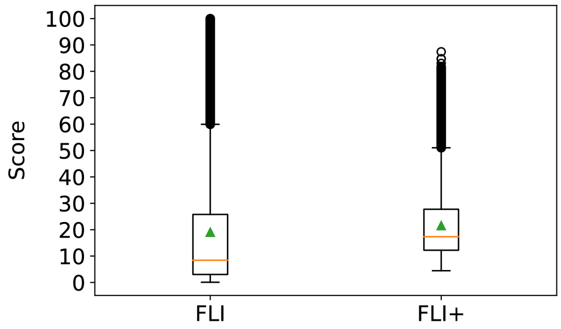

(c) Female subjects

Fig S8. **Box-plots of fatty liver index (FLI) and FLI+ using (a) all subjects; (b) male subjects; and (c) female subjects in a non-imaging UK Biobank cohort.**

Figure C

We extended our analysis of an additional dataset from a non-imaging cohort in the UK Biobank introduced in Section Text C by stratifying the data according to three risk groups of the fatty liver index (FLI) and FLI+, such that Figs S9(a) and (b) illustrates the distribution of the indices across all subjects; Figs S9(c) and (d) across male subjects and Figs S9(e) and (f) cross female subjects.

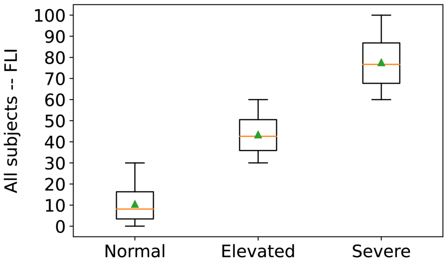

(a) FLI - All subjects

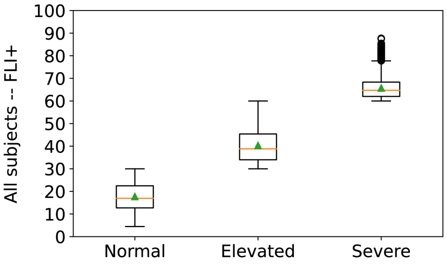

(b) FLI+ - All subjects

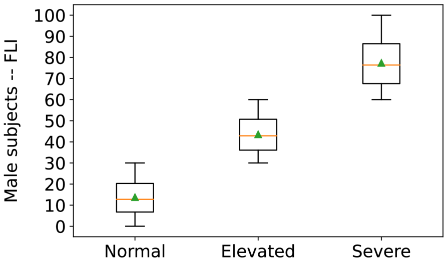

(c) FLI - Male subjects

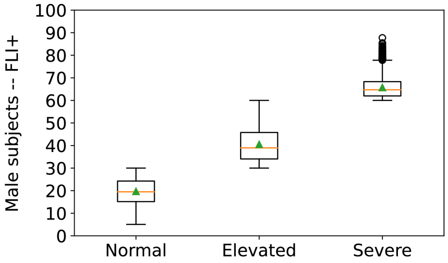

(d) FLI+ - Male subjects

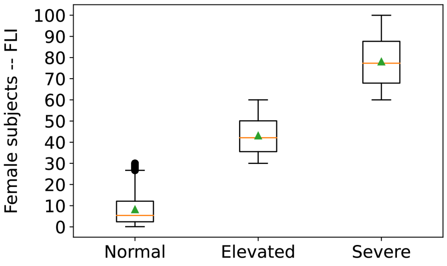

(e) FLI - Female subjects

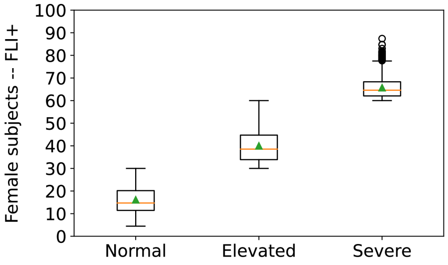

(f) FLI+ - Female subjects

Fig S9. **Box-plots of fatty liver index (FLI) and FLI+ stratified by three risk groups in all subjects (a)(b); male subjects (c)(d); and female subjects (e)(f) in a non-imaging UK Biobank cohort.**

References

1. Schneider CV, Zandvakili I, Thaiss CA, Schneider KM. Physical activity is associated with reduced risk of liver disease in the prospective UK Biobank cohort. JHEP Reports. 2021;3(3):100263. doi:10.1016/j.jhepr.2021.100263.
